# *N*,*N*′-Bis(2-hydroxy­ethyl)-*N*,*N*′-[ethyl­ene­dioxy­bis(*o*-phenyl­enemethyl­ene)]­diammonium fumarate tetra­hydrate

**DOI:** 10.1107/S1600536808033746

**Published:** 2008-10-22

**Authors:** Hong-Ye Bai, Hai-Yan Liu, Jian-Fang Ma

**Affiliations:** a Department of Chemistry, Northeast Normal University, Changchun 130024, People’s Republic of China

## Abstract

The reaction of 1,2-bis­{2-[(2-hydroxy­ethyl)amino­methyl]­phen­oxy}ethane and fumaric acid in a mixed solution in ethanol–water (1:1 *v*/*v*) yields the title compound, C_20_H_30_N_2_O_4_^2+^·C_4_H_2_O_4_^2−^·4H_2_O. In the crystal structure, the anions, cations and water mol­ecules are connected via O—H⋯O and N—H⋯O hydrogen bonds into a three-dimensional network. The fumarate anion and the *N*,*N*′-bis­(2-hydroxy­ethyl)-*N*,*N*′-[ethyl­enedioxy­bis(*o*-phenyl­enemethylene)]diammonium cation are located on centers of inversion, whereas the two crystallographically independent water mol­ecules occupy general positions.

## Related literature

For a related structure, see: Wang & Wei (2005 ▶). For background to the synthesis, see: Armstrong & Lindoy (1975 ▶).
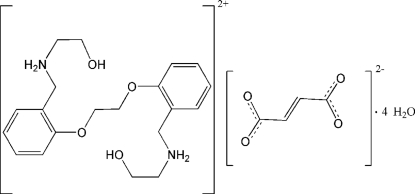


## Experimental

### 

#### Crystal data

C_20_H_30_N_2_O_4_^2+^·C_4_H_2_O_4_^2−^·4H_2_O
*M* *_r_* = 548.58
Triclinic, 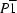

*a* = 7.585 (7) Å
*b* = 8.623 (6) Å
*c* = 11.515 (7) Å
α = 104.64 (2)°
β = 96.77 (3)°
γ = 104.29 (3)°
*V* = 693.0 (9) Å^3^
*Z* = 1
Mo *K*α radiation
μ = 0.11 mm^−1^
*T* = 293 (2) K
0.41 × 0.34 × 0.28 mm

#### Data collection

Rigaku R-AXIS RAPID diffractometer
Absorption correction: multi-scan (*ABSCOR*; Higashi, 1995 ▶) *T*_min_ = 0.952, *T*_max_ = 0.983
6826 measured reflections
3128 independent reflections
2394 reflections with *I* > 2σ(*I*)
*R*_int_ = 0.018

#### Refinement

*R*[*F* ^2^ > 2σ(*F* ^2^)] = 0.045
*wR*(*F* ^2^) = 0.128
*S* = 1.11
3128 reflections
193 parameters
H atoms treated by a mixture of independent and constrained refinement
Δρ_max_ = 0.68 e Å^−3^
Δρ_min_ = −0.20 e Å^−3^

### 

Data collection: *PROCESS-AUTO* (Rigaku, 1998 ▶); cell refinement: *PROCESS-AUTO*; data reduction: *PROCESS-AUTO*; program(s) used to solve structure: *SHELXS97* (Sheldrick, 2008 ▶); program(s) used to refine structure: *SHELXL97* (Sheldrick, 2008 ▶); molecular graphics: *SHELXTL* (Sheldrick, 2008 ▶); software used to prepare material for publication: *SHELXL97*.

## Supplementary Material

Crystal structure: contains datablocks global, I. DOI: 10.1107/S1600536808033746/nc2116sup1.cif

Structure factors: contains datablocks I. DOI: 10.1107/S1600536808033746/nc2116Isup2.hkl

Additional supplementary materials:  crystallographic information; 3D view; checkCIF report

## Figures and Tables

**Table 1:** Hydrogen-bond geometry (Å, °)

*D*—H⋯*A*	*D*—H	H⋯*A*	*D*⋯*A*	*D*—H⋯*A*
O1*W*—H1*C*⋯O2*W*	0.85 (3)	1.97 (3)	2.792 (3)	164 (3)
O2*W*—H2*C*⋯O2	0.85 (3)	1.97 (3)	2.828 (3)	176 (3)
N5—H5*B*⋯O2	0.950 (19)	1.811 (19)	2.749 (2)	168.6 (16)
O1*W*—H1*D*⋯O3^i^	0.91 (3)	1.96 (3)	2.830 (3)	159 (3)
O2*W*—H2*D*⋯O4^ii^	0.86 (3)	2.01 (3)	2.867 (3)	175 (3)
N5—H5*A*⋯O1*W*^iii^	0.900 (19)	2.125 (19)	2.925 (3)	147.6 (15)

Symmetry codes: (i) 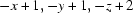; (ii) 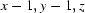; (iii) 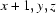.

**Fig. 1.**
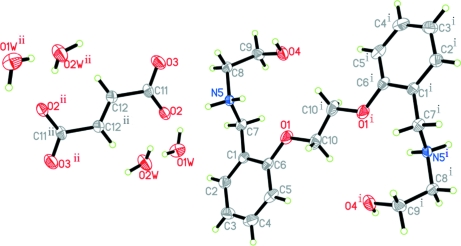
Crystal structure of the title compound with labelling and displacement ellipsoids drawn at the 30% probability level (for the H atoms spheres of arbitrary size are used). Symmetry codes: (i) -x + 2, -y + 1, -z + 1; (ii) -x + 1, -y, -z + 2.

**Fig. 2.**
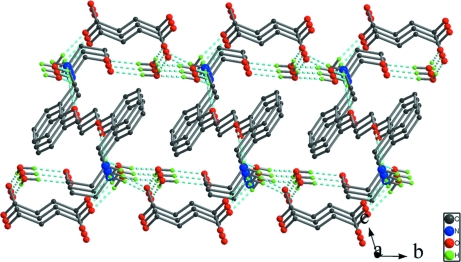
Crystal structure of the title compound with hydrogen bonding indicated by dashed lines. For clarity, H atoms not involved in hydrogen bonding are omitted.

## supplementary crystallographic information

### Comment

Currently, many groups are investigating crystal structures of cocrystals containing organic acids and organic bases, which are based on hydrogen bonding (Wang & Wei, 2005). In this context, the crystal structure of the title compound is presented.

The crystal structure consists of one N,N'-bis(2-hydroxyethyl)-N,N'-[ethylenedioxybis(o-phenylenemethylene)]diammonium cation and one fumarate anion located on centers of inversion and two crystallographically independent water molecules that occupy general positions (Fig. 1). In the crystal structure, different O—H···O and N—H···O hydrogen bonds link the components of the title compound into a three-dimensional network (Table 1 and Fig. 2).

### Experimental

All chemicals were obtained from commercial sources and used without further purification except for *L* (*L* is 1,2-bis{2-[(2-hydroxyethyl)aminomethyl]phenoxy}ethane). 1,4-bis(2-formylphenyl)-1,4-dioxabutuane, which was prepared according to the literature (Armstrong & Lindoy, 1975). 2-aminoethanol (1.22 g, 2.0 mol) in 50 ml of methanol were added slowly to a stirred boiling solution of 1,4-bis(2'-formylphenyl)-1,4-dioxabutuane (2.70 g, 1 mol) in 100 ml of methanol. The mixed solution was refluxed for 1 h, filtered off and cooled to room temperature. 1.52 g NaBH_4_ were added slowly to the filtrate and stirred for a further 2 h. The solvent was evaporated and 100 ml water of were added. After extraction with chloroform the solvent was evaporated, which led to a white solid of *L*. Crystals of the title compound were obtained by dissolving *L* (0.180 g, 1 mmol) and fumaric acid (0.116 g, 1 mmol) in a mixture of ethanol and water (1:1), followed by slow evaporation of the solvents.

### Refinement

The C—H H atoms were positioned with idealized geometry and treated as riding atoms with distances C—H = 0.97 (CH_2_) and 0.93 Å (CH).90 Å and *U*_iso_ = 1.2*U*_eq_(C). The H atoms bonded to N atoms and O atoms were located in a difference Fourier map and were refined vith varying coordinates isotropic with *U*_iso_(H) = 1.5*U*_eq_ of the parent atom.

### Crystal data

**Table:**

C_20_H_30_N_2_O_4_^2+^·C_4_H_2_O_4_^2^^−^·4H_2_O	*Z* = 1
*M**_r_* = 548.58	*F*(000) = 294
Triclinic, *P*1	*D*_x_ = 1.315 Mg m^−^^3^
Hall symbol: -P 1	Mo *K*α radiation, λ = 0.71069 Å
*a* = 7.585 (7) Å	Cell parameters from 3128 reflections
*b* = 8.623 (6) Å	θ = 3.1–27.4°
*c* = 11.515 (7) Å	µ = 0.11 mm^−^^1^
α = 104.64 (2)°	*T* = 293 K
β = 96.77 (3)°	Block, colourless
γ = 104.29 (3)°	0.41 × 0.34 × 0.28 mm
*V* = 693.0 (9) Å^3^	

### Data collection

**Table:**

Rigaku R-AXIS RAPID diffractometer	3128 independent reflections
Radiation source: fine-focus sealed tube	2394 reflections with *I* > 2σ(*I*)
graphite	*R*_int_ = 0.018
Detector resolution: 10.0 pixels mm^-1^	θ_max_ = 27.4°, θ_min_ = 3.1°
ω scans	*h* = −9→9
Absorption correction: multi-scan (ABSCOR; Higashi, 1995)	*k* = −11→10
*T*_min_ = 0.952, *T*_max_ = 0.983	*l* = −14→14
6826 measured reflections	

### Refinement

**Table:**

Refinement on *F*^2^	Primary atom site location: structure-invariant direct methods
Least-squares matrix: full	Secondary atom site location: difference Fourier map
*R*[*F*^2^ > 2σ(*F*^2^)] = 0.045	Hydrogen site location: inferred from neighbouring sites
*wR*(*F*^2^) = 0.128	H atoms treated by a mixture of independent and constrained refinement
*S* = 1.11	*w* = 1/[σ^2^(*F*_o_^2^) + (0.0619*P*)^2^ + 0.1182*P*] where *P* = (*F*_o_^2^ + 2*F*_c_^2^)/3
3128 reflections	(Δ/σ)_max_ < 0.001
193 parameters	Δρ_max_ = 0.68 e Å^−^^3^
0 restraints	Δρ_min_ = −0.20 e Å^−^^3^

### Special details

**Table:**

Geometry. All e.s.d.'s (except the e.s.d. in the dihedral angle between two l.s. planes) are estimated using the full covariance matrix. The cell e.s.d.'s are taken into account individually in the estimation of e.s.d.'s in distances, angles and torsion angles; correlations between e.s.d.'s in cell parameters are only used when they are defined by crystal symmetry. An approximate (isotropic) treatment of cell e.s.d.'s is used for estimating e.s.d.'s involving l.s. planes.
Refinement. Refinement of *F*^2^ against ALL reflections. The weighted *R*-factor *wR* and goodness of fit *S* are based on *F*^2^, conventional *R*-factors *R* are based on *F*, with *F* set to zero for negative *F*^2^. The threshold expression of *F*^2^ > σ(*F*^2^) is used only for calculating *R*-factors(gt) *etc*. and is not relevant to the choice of reflections for refinement. *R*-factors based on *F*^2^ are statistically about twice as large as those based on *F*, and *R*- factors based on ALL data will be even larger.

### Fractional atomic coordinates and isotropic or equivalent isotropic displacement parameters (Å^2^)

**Table:**

	*x*	*y*	*z*	*U*_iso_*/*U*_eq_	
C1	0.4884 (2)	0.33265 (18)	0.58122 (12)	0.0353 (3)	
C2	0.3124 (2)	0.2213 (2)	0.54836 (15)	0.0470 (4)	
H2	0.2257	0.2368	0.5977	0.056*	
C3	0.2632 (3)	0.0871 (2)	0.44313 (17)	0.0582 (5)	
H3	0.1438	0.0140	0.4212	0.070*	
C4	0.3923 (3)	0.0628 (2)	0.37144 (15)	0.0576 (5)	
H4	0.3599	−0.0284	0.3015	0.069*	
C5	0.5695 (3)	0.1718 (2)	0.40173 (14)	0.0507 (4)	
H5	0.6560	0.1539	0.3528	0.061*	
C6	0.6173 (2)	0.3083 (2)	0.50592 (12)	0.0388 (3)	
C7	0.5438 (2)	0.48184 (19)	0.69256 (13)	0.0381 (3)	
H7A	0.5907	0.5822	0.6688	0.046*	
H7B	0.4355	0.4914	0.7277	0.046*	
C8	0.7528 (2)	0.61530 (18)	0.90008 (13)	0.0406 (3)	
H8A	0.8408	0.5931	0.9574	0.049*	
H8B	0.6472	0.6266	0.9380	0.049*	
C9	0.8425 (2)	0.77871 (19)	0.87634 (15)	0.0471 (4)	
H9A	0.7507	0.8081	0.8269	0.056*	
H9B	0.8863	0.8665	0.9536	0.056*	
C10	0.9210 (2)	0.4234 (2)	0.46928 (15)	0.0532 (4)	
H10A	0.8711	0.4244	0.3880	0.064*	
H10B	0.9610	0.3235	0.4623	0.064*	
C11	0.6218 (2)	0.21555 (18)	0.97927 (15)	0.0403 (3)	
C12	0.5574 (2)	0.07174 (19)	1.03092 (14)	0.0431 (4)	
H12	0.6033	0.0873	1.1129	0.052*	
O1	0.78557 (15)	0.42777 (16)	0.54483 (10)	0.0503 (3)	
O2	0.52895 (15)	0.21994 (13)	0.88286 (10)	0.0485 (3)	
O3	0.76640 (19)	0.32235 (16)	1.03867 (13)	0.0684 (4)	
O1W	0.0354 (2)	0.3790 (2)	0.77573 (13)	0.0646 (4)	
H1C	0.050 (4)	0.289 (4)	0.787 (2)	0.097*	
H1D	0.122 (4)	0.462 (4)	0.836 (3)	0.097*	
O2W	0.1427 (2)	0.09631 (18)	0.79075 (15)	0.0672 (4)	
H2C	0.260 (5)	0.129 (4)	0.818 (3)	0.101*	
H2D	0.101 (4)	−0.004 (4)	0.795 (3)	0.101*	
O4	0.99312 (17)	0.76942 (15)	0.81566 (12)	0.0521 (3)	
H4A	1.073 (3)	0.739 (3)	0.860 (2)	0.078*	
N5	0.68936 (17)	0.46959 (14)	0.78706 (10)	0.0310 (3)	
H5A	0.792 (3)	0.458 (2)	0.7581 (16)	0.046*	
H5B	0.638 (2)	0.374 (2)	0.8117 (15)	0.046*	

### Atomic displacement parameters (Å^2^)

**Table:**

	*U*^11^	*U*^22^	*U*^33^	*U*^12^	*U*^13^	*U*^23^
C1	0.0385 (8)	0.0408 (8)	0.0311 (6)	0.0160 (6)	0.0048 (5)	0.0145 (6)
C2	0.0411 (9)	0.0564 (10)	0.0431 (8)	0.0116 (7)	0.0047 (7)	0.0178 (7)
C3	0.0580 (11)	0.0534 (10)	0.0505 (10)	0.0022 (9)	−0.0066 (8)	0.0145 (8)
C4	0.0831 (14)	0.0453 (9)	0.0381 (8)	0.0208 (9)	−0.0048 (8)	0.0062 (7)
C5	0.0675 (12)	0.0614 (10)	0.0326 (7)	0.0346 (9)	0.0104 (7)	0.0139 (7)
C6	0.0426 (8)	0.0501 (9)	0.0309 (7)	0.0206 (7)	0.0070 (6)	0.0172 (6)
C7	0.0407 (8)	0.0432 (8)	0.0363 (7)	0.0194 (6)	0.0082 (6)	0.0142 (6)
C8	0.0494 (9)	0.0363 (8)	0.0324 (7)	0.0077 (7)	0.0096 (6)	0.0077 (6)
C9	0.0546 (10)	0.0323 (8)	0.0485 (8)	0.0073 (7)	0.0042 (7)	0.0095 (7)
C10	0.0455 (9)	0.0832 (13)	0.0438 (9)	0.0271 (9)	0.0210 (7)	0.0269 (9)
C11	0.0413 (8)	0.0330 (7)	0.0523 (9)	0.0105 (6)	0.0112 (7)	0.0213 (7)
C12	0.0460 (9)	0.0398 (8)	0.0414 (8)	0.0063 (6)	0.0036 (6)	0.0166 (6)
O1	0.0404 (6)	0.0728 (8)	0.0381 (6)	0.0139 (6)	0.0152 (5)	0.0156 (5)
O2	0.0438 (6)	0.0429 (6)	0.0542 (7)	−0.0019 (5)	0.0034 (5)	0.0230 (5)
O3	0.0600 (8)	0.0487 (7)	0.0848 (10)	−0.0074 (6)	−0.0187 (7)	0.0361 (7)
O1W	0.0677 (9)	0.0684 (9)	0.0612 (8)	0.0352 (8)	0.0032 (6)	0.0141 (7)
O2W	0.0480 (8)	0.0537 (8)	0.1011 (11)	0.0065 (6)	−0.0001 (7)	0.0393 (8)
O4	0.0433 (7)	0.0515 (7)	0.0639 (7)	0.0049 (5)	0.0056 (5)	0.0319 (6)
N5	0.0327 (6)	0.0306 (6)	0.0313 (6)	0.0079 (5)	0.0087 (5)	0.0122 (5)

### Geometric parameters (Å, °)

**Table:**

C1—C2	1.383 (2)	C9—O4	1.416 (2)
C1—C6	1.400 (2)	C9—H9A	0.9700
C1—C7	1.499 (2)	C9—H9B	0.9700
C2—C3	1.385 (3)	C10—O1	1.424 (2)
C2—H2	0.9300	C10—C10^i^	1.492 (4)
C3—C4	1.374 (3)	C10—H10A	0.9700
C3—H3	0.9300	C10—H10B	0.9700
C4—C5	1.383 (3)	C11—O3	1.235 (2)
C4—H4	0.9300	C11—O2	1.257 (2)
C5—C6	1.389 (2)	C11—C12	1.508 (2)
C5—H5	0.9300	C12—C12^ii^	1.293 (3)
C6—O1	1.369 (2)	C12—H12	0.9300
C7—N5	1.499 (2)	O1W—H1C	0.85 (3)
C7—H7A	0.9700	O1W—H1D	0.91 (3)
C7—H7B	0.9700	O2W—H2C	0.85 (3)
C8—N5	1.494 (2)	O2W—H2D	0.86 (3)
C8—C9	1.513 (2)	O4—H4A	0.88 (3)
C8—H8A	0.9700	N5—H5A	0.900 (19)
C8—H8B	0.9700	N5—H5B	0.950 (19)
			
C2—C1—C6	118.93 (15)	H8A—C8—H8B	107.7
C2—C1—C7	122.15 (14)	O4—C9—C8	112.16 (13)
C6—C1—C7	118.89 (14)	O4—C9—H9A	109.2
C1—C2—C3	120.98 (17)	C8—C9—H9A	109.2
C1—C2—H2	119.5	O4—C9—H9B	109.2
C3—C2—H2	119.5	C8—C9—H9B	109.2
C4—C3—C2	119.45 (18)	H9A—C9—H9B	107.9
C4—C3—H3	120.3	O1—C10—C10^i^	105.74 (17)
C2—C3—H3	120.3	O1—C10—H10A	110.6
C3—C4—C5	121.00 (17)	C10^i^—C10—H10A	110.6
C3—C4—H4	119.5	O1—C10—H10B	110.6
C5—C4—H4	119.5	C10^i^—C10—H10B	110.6
C4—C5—C6	119.42 (17)	H10A—C10—H10B	108.7
C4—C5—H5	120.3	O3—C11—O2	125.21 (14)
C6—C5—H5	120.3	O3—C11—C12	114.98 (14)
O1—C6—C5	125.51 (15)	O2—C11—C12	119.81 (14)
O1—C6—C1	114.30 (14)	C12^ii^—C12—C11	124.47 (19)
C5—C6—C1	120.19 (16)	C12^ii^—C12—H12	117.8
N5—C7—C1	112.04 (12)	C11—C12—H12	117.8
N5—C7—H7A	109.2	C6—O1—C10	118.86 (13)
C1—C7—H7A	109.2	H1C—O1W—H1D	104 (2)
N5—C7—H7B	109.2	H2C—O2W—H2D	108 (3)
C1—C7—H7B	109.2	C9—O4—H4A	108.2 (15)
H7A—C7—H7B	107.9	C8—N5—C7	114.91 (12)
N5—C8—C9	113.42 (13)	C8—N5—H5A	106.3 (11)
N5—C8—H8A	108.9	C7—N5—H5A	112.5 (11)
C9—C8—H8A	108.9	C8—N5—H5B	106.9 (10)
N5—C8—H8B	108.9	C7—N5—H5B	107.6 (10)
C9—C8—H8B	108.9	H5A—N5—H5B	108.3 (15)
			
C6—C1—C2—C3	0.3 (2)	C2—C1—C7—N5	114.29 (16)
C7—C1—C2—C3	178.16 (14)	C6—C1—C7—N5	−67.81 (17)
C1—C2—C3—C4	1.1 (3)	N5—C8—C9—O4	−55.82 (19)
C2—C3—C4—C5	−1.0 (3)	O3—C11—C12—C12^ii^	−160.5 (2)
C3—C4—C5—C6	−0.3 (3)	O2—C11—C12—C12^ii^	19.9 (3)
C4—C5—C6—O1	−178.31 (14)	C5—C6—O1—C10	5.5 (2)
C4—C5—C6—C1	1.6 (2)	C1—C6—O1—C10	−174.42 (13)
C2—C1—C6—O1	178.34 (12)	C10^i^—C10—O1—C6	174.87 (16)
C7—C1—C6—O1	0.37 (18)	C9—C8—N5—C7	−61.95 (18)
C2—C1—C6—C5	−1.6 (2)	C1—C7—N5—C8	179.32 (12)
C7—C1—C6—C5	−179.58 (13)		

Symmetry codes: (i) −*x*+2, −*y*+1, −*z*+1; (ii) −*x*+1, −*y*, −*z*+2.

### Hydrogen-bond geometry (Å, °)

**Table:**

*D*—H···*A*	*D*—H	H···*A*	*D*···*A*	*D*—H···*A*
O1W—H1C···O2W	0.85 (3)	1.97 (3)	2.792 (3)	164 (3)
O2W—H2C···O2	0.85 (3)	1.97 (3)	2.828 (3)	176 (3)
N5—H5B···O2	0.950 (19)	1.811 (19)	2.749 (2)	168.6 (16)
O1W—H1D···O3^iii^	0.91 (3)	1.96 (3)	2.830 (3)	159 (3)
O2W—H2D···O4^iv^	0.86 (3)	2.01 (3)	2.867 (3)	175 (3)
N5—H5A···O1W^v^	0.900 (19)	2.125 (19)	2.925 (3)	147.6 (15)

Symmetry codes: (iii) −*x*+1, −*y*+1, −*z*+2; (iv) *x*−1, *y*−1, *z*; (v) *x*+1, *y*, *z*.
